# Deciphering the pathogenesis of sporadic Creutzfeldt-Jakob disease with codon 129 M/V and type 2 abnormal prion protein

**DOI:** 10.1186/2051-5960-1-74

**Published:** 2013-11-13

**Authors:** Atsushi Kobayashi, Yasushi Iwasaki, Hiroyuki Otsuka, Masahito Yamada, Mari Yoshida, Yuichi Matsuura, Shirou Mohri, Tetsuyuki Kitamoto

**Affiliations:** 1 Division of Neurological Science, Tohoku University Graduate School of Medicine, 2-1 Seiryo-machi, Aoba-ku, Sendai 980-8575, Japan; 2 Department of Neuropathology, Institute for Medical Science of Ageing, Aichi Medical University, 1-1 Yazakokarimata, Nagakute, Aichi 480-1195, Japan; 3 Otsuka Hospital, 513 Kinuyama, Hikami-cho, Tanba, Hyogo 669-3641, Japan; 4 Department of Neurology and Neurobiology of Ageing, Kanazawa University Graduate School of Medical Science, 13-1 Takara-machi, Kanazawa 920-8640, Japan; 5 Influenza and Prion Disease Research Center, National Institute of Animal Health, Tsukuba, Ibaraki 305-0856, Japan

**Keywords:** Creutzfeldt-Jakob disease, Prion protein, Classification, Humanized knock-in mouse

## Abstract

**Background:**

Sporadic Creutzfeldt-Jakob disease is classified according to the genotype at polymorphic codon 129 (M or V) of the prion protein (PrP) gene and the type (1 or 2) of abnormal isoform of PrP (PrP^Sc^) in the brain. The most complicated entity in the current classification system is MV2, since it shows wide phenotypic variations, *i.e.*, MV2 cortical form (MV2C), MV2 with kuru plaques (MV2K), or a mixed form (MV2K + C). To resolve their complicated pathogenesis, we performed a comprehensive analysis of the three MV2 subgroups based on histopathological, molecular, and transmission properties.

**Results:**

In histopathological and molecular analyses, MV2C showed close similarity to MM2 cortical form (MM2C) and could be easily discriminated from the other MV2 subgroups. By contrast, MV2K and MV2K + C showed the same molecular type and the same transmission type, and the sole difference between MV2K and MV2K + C was the presence of cortical pathology characteristic of MV2C/MM2C. The remarkable molecular feature of MV2K or MV2K + C was a mixture of type 2 PrP^Sc^ and intermediate type PrP^Sc^, which shows intermediate electrophoretic mobility between types 1 and 2 PrP^Sc^. Modeling experiments using PrP-humanized mice indicated that MV2K contains a mixture of intermediate type PrP^Sc^ with the 129M genotype (Mi PrP^Sc^) and type 2 PrP^Sc^ with the 129V genotype (V2 PrP^Sc^) that originated from V2 PrP^Sc^, whereas MV2C + K may also contain type 2 PrP^Sc^ with the 129M genotype and cortical pathology (M2C PrP^Sc^) that lacks infectivity to the PrP-humanized mice in addition to Mi and V2 PrP^Sc^.

**Conclusions:**

Taken together, the present study suggests that the phenotypic heterogeneity of MV2 stems from their different PrP^Sc^ origin(s).

## Background

Creutzfeldt-Jakob disease (CJD) is a lethal transmissible neurodegenerative disease caused by an abnormal isoform of prion protein (PrP^Sc^), which is converted from the normal cellular isoform (PrP^C^) [1]. There is a polymorphism (methionine, M; or valine, V) at codon 129 of the prion protein (PrP) gene. Parchi and colleagues reported that the codon 129 genotype (M/M, M/V, or V/V) and the type (type 1 or type 2) of PrP^Sc^ in the brain are major determinants of the clinicopathological phenotypes of sporadic CJD (sCJD) [2-5]. Type 1 and type 2 PrP^Sc^ are distinguishable according to the size of the proteinase K (PK)-resistant core of PrP^Sc^ (21 and 19 kDa, respectively), reflecting differences in the PK-cleavage site (at residues 82 and 97, respectively) [6]. According to this molecular typing system, sCJD is classified into six subgroups (MM1, MM2, MV1, MV2, VV1, or VV2). In addition, MM2 can be divided into two subgroups based on histopathological criteria (MM2C, cortical form showing a predominant cortical pathology; or MM2T, thalamic form showing characteristic atrophy of thalamic and inferior olivary nuclei) [4]. This Parchi’s classification based on genotyping, PrP^Sc^ typing, and histotyping, has been widely used compared with an alternative classification system proposed by others [7,8].

Among the seven sCJD subgroups, the most complicated entity is MV2, which accounts for 11% of total sCJD [5]. Since MV2 shows wide phenotypic variations, Parchi and colleagues have proposed dividing this heterogeneous entity based on histopathological criteria (MV2C showing a predominant cortical pathology or MV2K showing kuru type PrP amyloid plaques) [9]. Moreover, the co-occurrence of these histotypes in the same brain (denoted as MV2K + C) has also been reported [9]. MV2K is the most common subgroup in MV2 (8% of total sCJD cases), whereas MV2C is very rare (< 0.5%) [5,9]. MV2K + C is relatively common but sometimes misdiagnosed as MV2K even by experts of CJD surveillance centers [10]. Besides these complicated histotypes, type 2 PrP^Sc^ in certain MV2 cases shows atypical features, *i.e.*, wide, heterogeneous fragments that migrate at approximately 20 to 19 kDa and are sometimes visible as doublets [4,11,12]. The atypical type 2 PrP^Sc^ has been reported in MV2K and MV2K + C but not in MV2C. The molecular mechanisms of the atypical type 2 PrP^Sc^ formation remain elusive. In addition, the atypical type 2 PrP^Sc^ has not been recapitulated in an experimental transmission of MV2K to PrP-humanized mice carrying the 129M/V genotype [13].

To characterize the MV2 subgroups in detail and to resolve their complicated pathogenesis, we performed a comprehensive analysis of the MV2 subgroups based on histopathological, molecular and transmission properties.

## Methods

### Patients

CJD cases included in this study were patients with clinically, genetically and histopathologically proven sCJD. Brain tissues were obtained at autopsy from CJD patients after receiving informed consent for research use. The diagnosis of sCJD, histotype, and PrP^Sc^ type were confirmed by PrP immunohistochemistry and western blot analysis [14,15]. The genotype and the absence of mutations in the open reading frame of the PrP gene were determined by sequence analysis as described [16]. According to Parchi’s classification [4,5], these sCJD cases were classified as follows: MM1, 1 case; MM2C, 1 case; VV2, 1 case; MV2C, 1 case; MV2K, 1 case; and MV2K + C, 1 case. These patients showed the typical phenotypes of each sCJD subgroup in the clinicopathological and biochemical examinations. Ethical approval for these studies was obtained from the Ethical Committee of Tohoku University Graduate School of Medicine. All experiments carried out on humans were in compliance with the Helsinki Declaration.

### Transmission experiments

Brain homogenates (10%) were prepared as described [17]. Intracerebral transmission to PrP-humanized mice was performed using 20 μl of the homogenates [15]. The production of the knock-in mice expressing human PrP with the 129M/M genotype (Ki-Hu129M/M), Ki-Hu129M/V, and Ki-Hu129V/V has been reported [17,18]. The expression levels of human PrP in the brains of these knock-in mice were identical to the level observed in wild-type mouse. The inoculated mice were sacrificed after the onset of disease or at death. One hemisphere of the brain was fixed in 10% buffered formalin for immunohistochemistry, and the other hemisphere was immediately frozen for western blotting. Ethical approval for these experiments using mice was obtained from Animal Care and Use Committee of Tohoku University Graduate School of Medicine.

### Immunohistochemistry

Formalin-fixed brain tissues were treated with formic acid (99% for human tissues or 60% for mouse tissues) for 1 hour to inactivate the infectivity, and embedded in paraffin. Tissue sections were pretreated by hydrolytic autoclaving before PrP immunohistochemistry [14]. The anti-PrP monoclonal antibodies 3F4 [19] and #71 [20,21] were used as the primary antibodies for human sections, and anti-PrP antiserum PrP-N [22] was used as the primary antibody for mouse sections. Goat-anti-mouse immunoglobulin polyclonal antibody labelled with the peroxidase-conjugated dextran polymer, EnVision + (Dako) and anti-rabbit EnVision + were used as the secondary antibodies. For the quantification of PrP plaques in the patient brains, at least six representative digital microscopy images were taken at 10× magnification from each brain region and analyzed using ImageJ software (rsb.info.nih.gov/ij). The number of PrP plaques was manually counted, and the mean plaque density in each brain region was calculated.

### Western blotting

PrP^Sc^ was extracted from human or mouse brains with collagenase treatment as described [23]. For deglycosylation of PrP^Sc^, samples were digested with PNGaseF (New England Biolabs) as reported [24]. Protein samples were subjected to SDS-PAGE using 15% Tris-glycine long gels of 15 cm length and western blotting [18]. Type 1 PrP^Sc^- and type 2 PrP^Sc^-specific polyclonal antibodies (designated as Tohoku 1 (T1) and Tohoku 2 (T2), respectively [25]) and the 3F4 antibody were used as the primary antibodies. Anti-rabbit EnVision + and anti-mouse EnVision + were used as the secondary antibodies. The signal intensities of the western blots were quantified with Quantity One software using an imaging device VersaDoc 5000 (Bio-Rad Laboratories).

### Statistical analysis

Incubation times and the signal intensities of PrP^Sc^ bands are expressed as mean ± SEM.

## Results

Histotyping and molecular typing of MV2.

**Figure 1:**
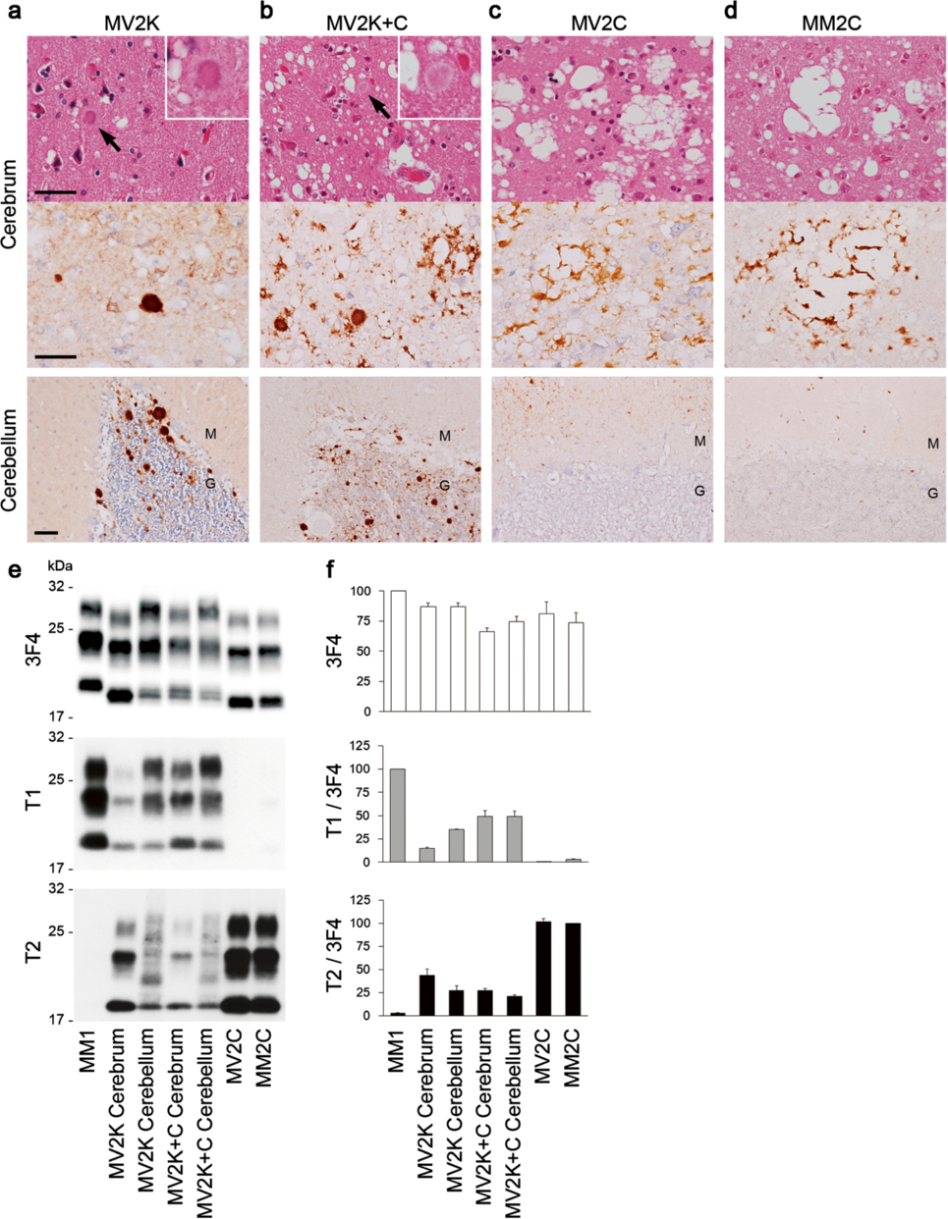
**Histotyping and molecular typing of the MV2 subgroups. (a-d)** Histopathological features of MV2K **(a)**, MV2K + C **(b)**, MV2C **(c)**, or MM2C **(d)** (hematoxylin and eosin stain and immunohistochemistry for PrP). Higher magnification of kuru plaques (arrows) is shown in the insets. M, molecular layer; G, granular cell layer. Scale bar: 100 μm. **(e)** Western blot analysis of PrP^Sc^ in the brains using anti-PrP antibody 3F4 and PrP^Sc^-type specific antibodies T1 or T2. Protein samples were prepared from the frontal cortices. In addition, cerebellar cortices of MV2K or MV2K + C were also examined. **(f)** The mean signal intensities of 3F4-, T1-, or T2-reactive PrP^Sc^. To compare the amounts of the T1- or T2-reactive PrP^Sc^ among the patients, the signal intensities of the T1- or T2-reactive PrP^Sc^ were normalized by those of the 3F4-reactive PrP^Sc^. The mean signal intensities of MM1 were assigned as 100 /mm^2^ in each experiment using 3F4 or T1 antibody, and those of MM2C were assigned as 100 /mm^2^ in each experiment using T2 antibody. All experiments were repeated independently at least three times.

At first, we performed histopathological analysis of the three subgroups of MV2 (MV2K, MV2K + C, and MV2C; Parchi’s classification [4,5] is used in this paper). MV2K showed unicentric PrP amyloid plaques, kuru plaques, throughout the cerebral grey matter and the cerebellum as reported previously (Figure 1a, Additional file 1: Figure S1a, e) [4]. These plaques were prominent especially in the granular cell layer of the cerebellum. MV2K + C showed large confluent vacuoles and perivacuolar PrP deposition in the cerebral cortices besides scattered kuru plaques as reported (Figure 1b, Additional file 1: Figure S1b, f) [4]. The large confluent vacuoles and perivacuolar PrP deposition in the cerebral cortices were characteristics of MV2C and MM2C (Figure 1c, d; Additional file 1: Figure S1c, d, g, h) [4,9]. The cerebellum of MV2K + C contained numerous PrP plaques in the granular cell layer. Thus, MV2K + C showed mixed histotypes, *i.e.*, a mixture of the MV2C/MM2C phenotype and MV2K phenotype. PrP plaques were absent in the brain of MV2C even in the granular cell layer of the cerebellum. Instead, MV2C and MM2C showed focal amorphous patchy PrP deposition in the molecular layer of the cerebellum as reported [5]. Thus, the three MV2 subgroups exhibited clearly different histotypes.

Next we performed western blot analysis of PrP^Sc^ in the brains of three MV2 subgroups using PrP^Sc^ type-specific antibodies and a conventional anti-PrP antibody 3F4 that detects all PrP^Sc^ types. Type 1 PrP^Sc^-specific antibody Tohoku 1 (T1) recognizes epitopes between residues 82 and 96 that are retained in type 1 PrP^Sc^ but not in type 2 PrP^Sc^ after PK-digestion. In addition, the epitopes for the T1 antibody are also retained in the intermediate type PrP^Sc^ showing intermediate electrophoretic mobility between types 1 and 2 PrP^Sc^, which is observed in a part of dura mater-graft associated CJD [25]. Type 2 PrP^Sc^-specific antibody Tohoku 2 (T2) specifically detects the N-terminal PK-cleavage site of type 2 PrP^Sc^ (at residue 97) [25]. In the conventional western blot analysis using the anti-PrP antibody 3F4, MV2K and MV2K + C had heterogeneous unglycosylated bands located at 20 kDa to 19 kDa as reported (Figure 1e) [4]. Since the upper unglycosylated band migrated faster compared with type 1 PrP^Sc^ from MM1, these heterogeneous bands were different from the co-occurrence of types 1 (21 kDa) and 2 (19 kDa) PrP^Sc^. In contrast, MV2C and MM2C had only type 2 PrP^Sc^ located at 19 kDa. In the western blot analysis using the PrP^Sc^ type-specific antibodies, MV2K and MV2K + C had large amounts of T1-reactive PrP^Sc^ in addition to T2-reactive PrP^Sc^ (Figure 1f). The amounts of T1-reactive PrP^Sc^ in the MV2K cerebrum were relatively low compared with the cerebellum, suggesting regional variability in the ratio of the amounts of T1-reactive PrP^Sc^ to the amounts of T2-reactive PrP^Sc^. The low amounts of T1-reactive PrP^Sc^ in the MV2K cerebrum accounted for the absence of the 20 kDa (upper) unglycosylated band in the western blot using the 3F4 antibody, since conventional western blot analysis using antibodies that react with all PrP^Sc^ types cannot detect a coexisting minority component [26]. In addition, the T2-reactive PrP^Sc^ in the cerebellum of MV2K or MV2K + C consisted of ladder-like multiple fragments, which were quite different from the T2-reactive PrP^Sc^ in the cerebrum. Thus, MV2K and MV2K + C showed similar molecular properties in the western blot analysis using the PrP^Sc^ type-specific antibodies. By contrast, MV2C and MM2C had predominant T2-reactive PrP^Sc^ with only trace amounts of T1-reactive PrP^Sc^ as reported [26,27]. MM1 had predominant T1-reactive PrP^Sc^ with trace amounts of T2-reactive PrP^Sc^ as reported [28]. Taken together, MV2C was clearly different from the other MV2 subgroups in the molecular typing as well, whereas MV2K and MV2K + C could not be distinguished by molecular typing even with the PrP^Sc^ type-specific antibodies.

### Transmission typing of MV2

**Figure 2:**
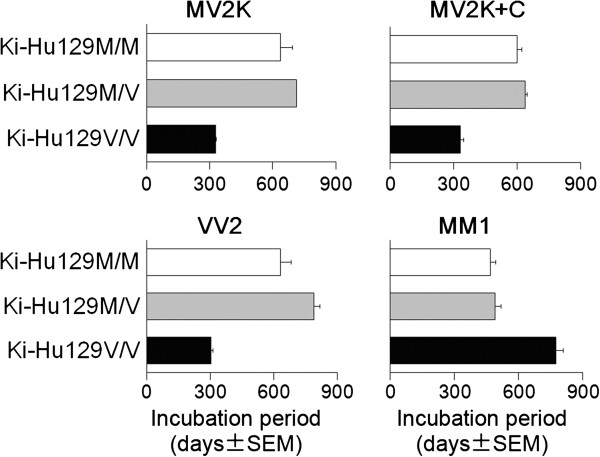
**Transmission of sCJD to PrP-humanized mice.** The mean incubation times of MV2K-inoculated Ki-Hu129M/M, Ki-Hu129M/V, and Ki-Hu129V/V were 638 ± 57 days (number of mice positive for PrP accumulation in immunohistochemical analysis/number of inoculated mice = 4/4), 713 ± 0 days (3/4), and 329 ± 3 days (4/4), respectively. The mean incubation times of MV2K + C-inoculated Ki-Hu129M/M, Ki-Hu129M/V, and Ki-Hu129V/V were 600 ± 22 days (6/6), 638 ± 11 days (7/7), and 332 ± 15 days (4/4), respectively. The mean incubation times of VV2-inoculated Ki-Hu129M/M, Ki-Hu129M/V, and Ki-Hu129V/V were 633 ± 49 days (6/6), 788 ± 30 days (4/4), and 302 ± 9 days (7/7), respectively. The mean incubation times of MM1-inoculated Ki-Hu129M/M, Ki-Hu129M/V, and Ki-Hu129V/V were 467 ± 24 days (8/8), 490 ± 26 days (5/5), and 774 ± 32 days (6/6), respectively. The details of the transmission experiments of VV2 or MM1 using Ki-Hu129M/M and Ki-Hu129V/V have been reported previously [25,29].

**Figure 3:**
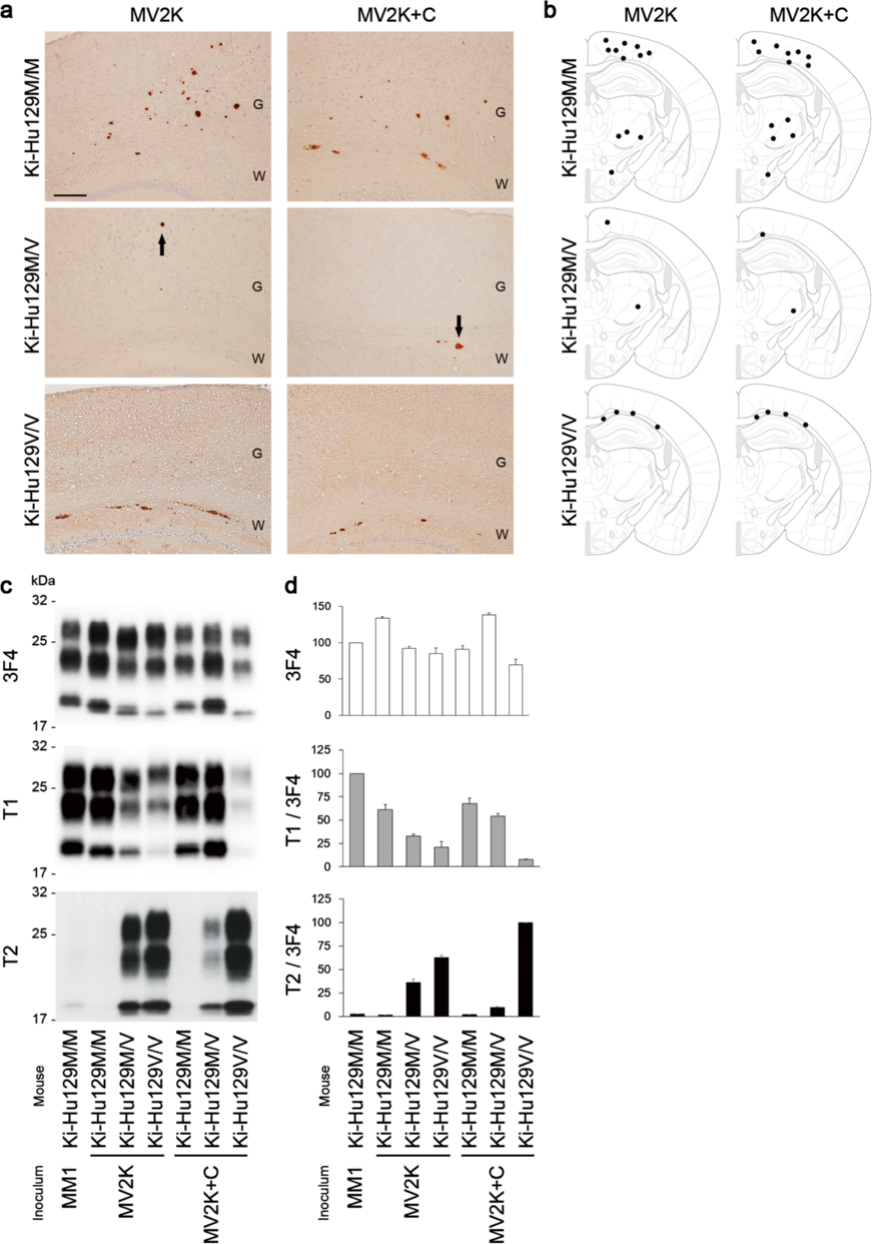
**Transmission properties of MV2K and MV2K + C. (a)** Immunohistochemical analysis of PrP in the brains of MV2K- or MV2K + C-inoculated mice. Ki-Hu129M/V showed fewer PrP plaques (arrows) compared with Ki-Hu129M/M. G, grey matter; W, white matter. Scale bar: 100 μm. **(b)** Summary of the distribution of PrP plaques in the brains of MV2K- or MV2K + C-inoculated mice. The distribution of PrP plaques (black dots) was plotted on the illustrations showing the level of bregma −1.79 mm from “The Mouse Brain, 4^th^ edition” [30]. **(c)** Western blot analysis of PrP^Sc^ in the brains using anti-PrP antibody 3F4 and PrP^Sc^-type specific antibodies T1 or T2. **(d)** The mean signal intensities of 3F4-, T1-, or T2-reactive PrP^Sc^. To compare the amounts of the T1- or T2-reactive PrP^Sc^ among the mice, the signal intensities of the T1- or T2-reactive PrP^Sc^ were normalized by those of the 3F4-reactive PrP^Sc^. The mean signal intensities of MM1-inoculated Ki-Hu129M/M were assigned as 100 /mm^2^ in each experiment using 3F4 or T1 antibody, and those of MV2K + C-inoculated Ki-Hu129V/V were assigned as 100 /mm^2^ in each experiment using T2 antibody. All experiments were repeated independently at least three times.

To characterize further the MV2 subgroups, we then performed transmission experiments using PrP-humanized mice. These knock-in mice (Ki-Hu129M/M, Ki-Hu129M/V, or Ki-Hu129V/V) express human PrP with either the 129M/M, M/V, or V/V genotype at the same level. Since the expression level of PrP affects the susceptibility to PrP^Sc^ infection regardless of the PrP genotype, these knock-in mice have an advantage over transgenic mice for evaluating the susceptibility among the genotypes [18]. After intracerebral challenge with the MV2K brain material, Ki-Hu129M/M and Ki-Hu129M/V showed much longer incubation times compared with those of Ki-Hu129V/V (Figure 2). Inoculation of the MV2K + C brain material resulted in the same transmission patterns as those of MV2K. Furthermore, the histopathological phenotypes of MV2K and those of MV2K + C in the inoculated mice were also indistinguishable (Figure 3a, b). Ki-Hu129M/M showed variously sized, round, plaque-type PrP deposits throughout the cerebral grey matter, white matter, striatum, and thalamus. Ki-Hu129M/V had fewer PrP plaques compared with the Ki-Hu129M/M. In Ki-Hu129V/V, PrP plaques were restricted to within the white matter. Large confluent vacuoles or perivacuolar PrP deposition, which are characteristics of MV2C/MM2C, were absent in the MV2K + C-inoculated mice. The conventional western blot analysis of PrP^Sc^ in the brains from MV2K- or MV2K + C-inoculated mice using the 3F4 antibody revealed that Ki-Hu129M/M produced the 20 kDa PrP^Sc^, Ki-Hu129M/V produced the atypical type 2 PrP^Sc^, *i.e.*, a mixture of the 20 kDa PrP^Sc^ and type 2 PrP^Sc^, and Ki-Hu129V/V produced type 2 PrP^Sc^ (Figure 3c). Western blot analysis using the PrP^Sc^ type-specific antibodies revealed that the amounts of T1-reactive PrP^Sc^ gradually decreased from Ki-Hu129M/M to Ki-Hu129M/V to Ki-Hu129V/V, while the amounts of T2-reactive PrP^Sc^ gradually increased from Ki-Hu129M/M to Ki-Hu129M/V to Ki-Hu129V/V (Figure 3d). Thus, MV2K and MV2K + C could also not be distinguished by transmission typing.

### Modelling of MV2

To resolve the complicated pathogenesis of MV2, we hypothesized that MV2K might have originated from type 2 PrP^Sc^ with the 129V genotype (denoted as V2 PrP^Sc^) and might contain V2 PrP^Sc^ and the intermediate type PrP^Sc^ with the 129M genotype (denoted as Mi PrP^Sc^), since we previously found that PrP^C^ with the 129M genotype in Ki-Hu129M/M converted into Mi PrP^Sc^ after challenge with VV2 brain material containing V2 PrP^Sc^. These Mi PrP^Sc^ showed striking similarity to the 20 kDa PrP^Sc^ in MV2K or MV2K + C [25,29,31]. Meanwhile, MV2K + C might also contain type 2 PrP^Sc^ with the 129M genotype and cortical pathology (denoted as M2C PrP^Sc^) in addition to Mi and V2 PrP^Sc^, since MV2C showed close similarity to MM2C that contains M2C PrP^Sc^, as described above.

**Figure 4:**
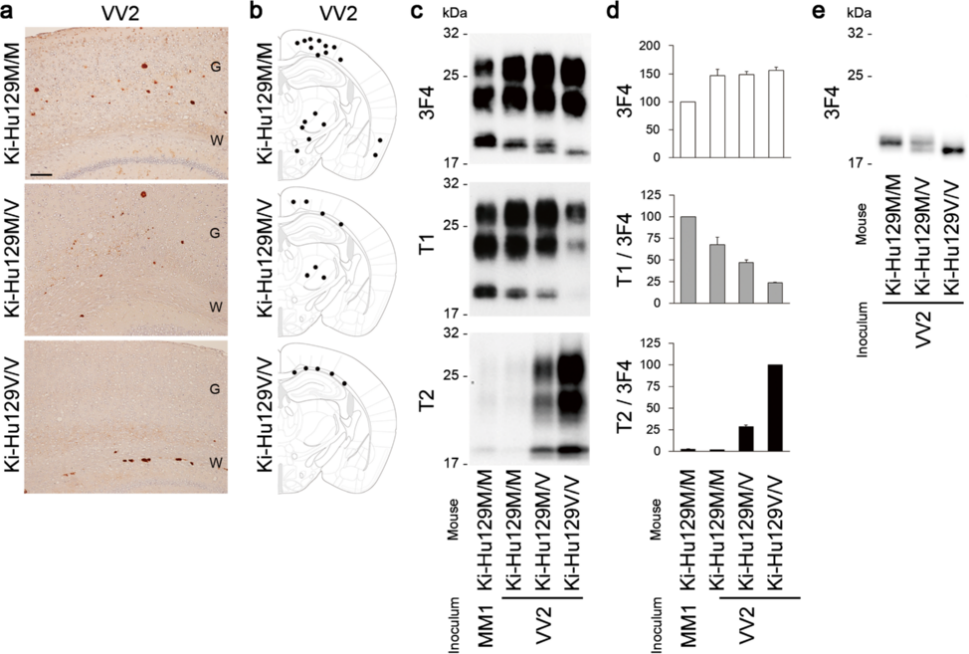
**Modelling of MV2K. (a)** Immunohistochemical analysis of PrP in the brains of VV2-inoculated PrP-humanized mice. G, grey matter; W, white matter. Scale bar: 100 μm. **(b)** Summary of the distribution of PrP plaques in the brains of VV2-inoculated mice. The distribution of PrP plaques (black dots) was plotted on the illustrations showing the level of bregma −1.79 mm from “The Mouse Brain, 4^th^ edition” [30]. **(c)** Western blot analysis of PrP^Sc^ in the brains using the anti-PrP antibody 3F4 and the PrP^Sc^-type specific antibodies T1 or T2. **(d)** The mean signal intensities of 3F4-, T1-, or T2-reactive PrP^Sc^. To compare the amounts of the T1- or T2-reactive PrP^Sc^ among the mice, the signal intensities of the T1- or T2-reactive PrP^Sc^ were normalized by those of the 3F4-reactive PrP^Sc^. The mean signal intensities of MM1-inoculated Ki-Hu129M/M were assigned as 100 /mm^2^ in each experiment using the 3F4 or T1 antibody, and those of VV2-inoculated Ki-Hu129V/V were assigned as 100 /mm^2^ in each experiment using the T2 antibody. All experiments were repeated independently at least three times. **(e)** Western blot analysis of deglycosylated PrP^Sc^ using the 3F4 antibody. Ki-Hu129M/M produced the intermediate type PrP^Sc^ located at 20 kDa, Ki-Hu129V/V produced type 2 PrP^Sc^ located at 19 kDa, and Ki-Hu129M/V produced a mixture of these PrP^Sc^ bands.

To test this hypothesis, we first performed intracerebral inoculation of a VV2 brain material containing V2 PrP^Sc^ into Ki-Hu129M/V, as an experimental model of MV2K. The VV2-inoculated Ki-Hu129M/V developed disease after a long incubation period (Figure 2). These mice showed PrP plaques in the cerebral grey matter, white matter, striatum, and thalamus (Figure 4a, b). As reported previously [29], VV2-inoculated Ki-Hu129M/M had more PrP plaques throughout the brain, whereas VV2-inoculated Ki-Hu129V/V showed restricted plaque distribution within the white matter. Western blot analysis of PrP^Sc^ in the brain revealed that VV2-inoculated Ki-Hu129M/V produced a mixture of the intermediate type PrP^Sc^ and type 2 PrP^Sc^ similar to the atypical type 2 PrP^Sc^ in the MV2K or MV2K + C patients (Figure 4c-e). VV2-inoculated Ki-Hu129M/M produced the intermediate type PrP^Sc^ with the 129M genotype (Mi PrP^Sc^), whereas VV2-inoculated Ki-Hu129V/V produced type 2 PrP^Sc^ with the 129V genotype (V2 PrP^Sc^) as reported [25,29]. These results indicated that V2 PrP^Sc^ generated both Mi and V2 PrP^Sc^ in animals with the 129M/V genotype.

Next we performed intracerebral inoculation of MM2C brain material containing M2C PrP^Sc^ into the PrP-humanized mice to gain insight into the reason why MV2K + C (containing M2C, Mi, and V2 PrP^Sc^) and MV2K (containing Mi and V2 PrP^Sc^) could not be distinguished by transmission typing. As expected, none of the MM2C-inoculated mice had developed disease by the end of their life span. An accumulation of PrP^Sc^ was absent in the brains of the MM2C-inoculated mice (Additional file 2: Figure S2). Thus, M2C PrP^Sc^ lacked infectivity to the PrP-humanized mice.

## Discussion

We performed a comprehensive analysis of the MV2 subgroups, *i.e.*, MV2C, MV2K, and MV2K + C, and resolved their complicated pathogenesis. MV2C showed close similarity to MM2C in the histopathological and molecular analyses and could be easily discriminated from the other MV2 subgroups. By contrast, MV2K and MV2K + C showed the same molecular type and the same transmission type. The remarkable molecular feature of MV2K or MV2K + C was a mixture of the intermediate type PrP^Sc^ and type 2 PrP^Sc^. To model MV2K, we inoculated VV2 brain material containing V2 PrP^Sc^ into the PrP-humanized mice with the 129M/V genotype. These mice showed widespread PrP plaques and the accumulation of a mixture of the intermediate type PrP^Sc^ with the 129M genotype (Mi PrP^Sc^) and V2 PrP^Sc^ in the brain. These results suggest that MV2K contains Mi and V2 PrP^Sc^ that originated from V2 PrP^Sc^. In addition, we also inoculated MM2C brain material containing M2C PrP^Sc^ into the PrP-humanized mice to gain insight into the reason why MV2K and MV2K + C could not be distinguished by transmission typing. These transmission experiments revealed that M2C PrP^Sc^ lacked infectivity to the PrP-humanized mice. Therefore, the present data lead us to surmise that MV2K + C may also contain M2C PrP^Sc^ lacking infectivity to the PrP-humanized mice in addition to Mi and V2 PrP^Sc^. Further study will be needed to verify that MV2C and MM2C are identical in transmission typing as well as histotyping and molecular typing. To grasp the whole picture of the phenotypic variability and PrP^Sc^ strain diversity in the MV2 subgroups, a transmission study of MV2C is in progress using the PrP-humanized mice.

The present study solves the mystery of the atypical type 2 PrP^Sc^ in the MV2 subgroups. This heterogeneous PrP^Sc^ consists of a mixture of the intermediate type PrP^Sc^ and type 2 PrP^Sc^, denoted as types i + 2. Differentiation of the intermediate type PrP^Sc^ and type 2 PrP^Sc^ is difficult with conventional western blot analysis and requires strict conditions for PK-digestion and high resolution gel electrophoresis systems, *e.g.*, 10-20% gradient Tris-glycine long gels or Bis-tris long gels (Figure 4e) [11]. However, they could be easily distinguished by the PrP^Sc^ type-specific antibodies in the present study. Moreover, the co-occurrence of the intermediate type PrP^Sc^ and type 2 PrP^Sc^ was recapitulated in the animal model for the first time. Parchi and colleagues once proposed to designate the atypical type 2 as type 2^+20kDa^[12], but they have never used this nomenclature thereafter. To characterize MV2 cases more adequately, we propose refined nomenclature as follows: MV2C, MVi + 2, or MVi + 2C (Table 1). The present study suggests that pure type 2 PrP^Sc^ in MV2C and the mixture of the intermediate type PrP^Sc^ and type 2 PrP^Sc^ in MVi + 2 or MVi + 2C are different and should be considered as distinct entities. In fact, Mi PrP^Sc^ is always accompanied by kuru plaques in MVi + 2, MVi + 2C, or dura mater graft-associated CJD with kuru plaques (p-dCJD) [29,32], suggesting that Mi PrP^Sc^ is a component of kuru plaques and is directly associated with the neuropathological phenotype. The amounts of Mi PrP^Sc^ might vary among brain regions, as shown in the western blot analysis of the MVi + 2 brain in the present study. Unfortunately, we could not test this possibility in detail because additional brain materials were not available. However, in the histopathological analysis, the abundance of kuru plaques varied among brain regions in the MVi + 2 or MVi + 2C patient (Additional file 1: Figure S1). The prominent kuru plaque formation in the cerebellum of MVi + 2 or MVi + 2C may indicate that histopathological and molecular analyses of the cerebellum would be useful to detect Mi PrP^Sc^ and kuru plaques, which are two phenotypic hallmarks of MVi + 2 or MVi + 2C. Meanwhile, even in our comprehensive analysis, the sole difference between MVi + 2 and MVi + 2C was the presence of MV2C/MM2C pathology. Since MVi + 2C cases with the very focal MV2C/MM2C pathology can be misdiagnosed as MVi + 2 [10], further study will be needed to assess whether MVi + 2 and MVi + 2C are distinguishable in their clinical features. Although we believe that the refined nomenclature proposed here would contribute to clarifying the complicated issues around the MV2 subgroups, the number of MV2 cases included in the present study was very limited because MV2C, MVi + 2, and MVi + 2C are rare in Japan compared with Western countries, reflecting the very low prevalence of the 129M/V genotype in the healthy population [33]. We analyzed a total of 232 cases with sCJD and found only 5 cases with MVi + 2, 2 cases with MV2C (one of the patients carried the M232R mutation on the 129M allele), and 1 case with MVi + 2C. Although typical cases of each subgroup were selected for the present study, we cannot exclude the possibility of phenotypic variation within each subgroup. Therefore, more extensive analyses of MV2 cases using PrP^Sc^ type-specific antibodies and transmission studies including additional MV2 cases are essential to validate the present findings. Of note, although several type 1 PrP^Sc^-specific antibodies have been reported [25-27], the amounts of detectable Mi PrP^Sc^ may differ among antibodies depending on their epitopes, since the epitopes located near the N-terminal PK-cleavage site of type 1 PrP^Sc^ can be lacking in Mi PrP^Sc^[25].

**Table 1:** **Refined nomenclature for the MV2 subgroups**^**a**^

				**Proposed nomenclature**						
	**Current classification**^**b**^	**PrP**^**Sc **^**type**	**Transmission type**^**c**^	**Classification**	**Codon 129 genotype**	**PrP**^**Sc **^**type**	**Histo-type**^**d**^	**Transmission type**	**Original PrP**^**Sc **^**strain(s)**	**Existing PrP**^**Sc **^**strain(s)**
Pure phenotypes	MM1	1	M1^CJD^	**MM1**	**MM**	**1**	**S**	**M1**	**M1**	**M1**
	MM2C	2	M2^CJD^	**MM2C**	**MM**	**2**	**C**	**―**	**M2C**	**M2C**
	MV2C	2	N.D.^e^	**MV2C**	**MV**	**2**	**C**	**N.D.**^**f**^	**M2C**	**M2C**
	MV2K	2	V2^CJD^	**MVi + 2**	**MV**	**i + 2**^**g**^	**K**	**V2**	**V2**	**Mi + V2**
	VV2	2	V2^CJD^	**VV2**	**VV**	**2**	**P**	**V2**	**V2**	**V2**
Mixed phenotype	MV2K + C	2	N.D.	**MVi + 2C**	**MV**	**i + 2**	**K + C**	**V2**	**M2C + V2**	**M2C + Mi + V2**

^a^Proposed nomenclature for the classification of sCJD subgroups analyzed in this study.

^b^According to Parchi *et al*., 1999 [4]; and Parchi *et al*., 2011 [5].

^c^According to Bishop *et al*., 2010 [13].

^d^S, synaptic; C, cortical form; K, kuru plaques; P, plaque-like.

^e^N.D., not done.

^f^Transmission experiment is in progress.

^g^Mixture of the intermediate type and type 2 PrP^Sc^.

The generation of multiple PrP^Sc^ strains in heterozygotes of the PrP genotypes can result in a long incubation period due to interference among coexisting heterologous PrP^Sc^, designated as heterozygous inhibition [34,35]. In the present study, V2 PrP^Sc^-inoculated Ki-Hu129M/V produced Mi and V2 PrP^Sc^ and showed longer incubation times compared with those of Ki-Hu129M/M despite its expression of PrP^C^ with the 129V genotype, which is an optimal substrate for V2 PrP^Sc^. Therefore, the long clinical course of MVi + 2 or MVi + 2C [5] may also be due to heterozygous inhibition among the coexisting Mi and V2 (and M2C) PrP^Sc^.

Despite the coexistence of Mi and V2 PrP^Sc^, MVi + 2 showed the same transmission properties as those of VV2 containing V2 PrP^Sc^ alone, as reported [13]. This result is consistent with our previous findings that Mi PrP^Sc^ was originated from V2 PrP^Sc^ and showed the same transmission properties as those of the parental V2 PrP^Sc^[25]. Meanwhile, MVi + 2C containing M2C, Mi, and V2 PrP^Sc^ also showed the same transmission properties as those of VV2, since M2C PrP^Sc^ lacked infectivity to the PrP-humanized mice and did not affect the transmission properties of the coexisting Mi and V2 PrP^Sc^. Thus, the transmission type does not reflect all existing PrP^Sc^ strains if their origins are identical or if they lack infectivity to experimental animals. Nevertheless, transmission typing will remain indispensable for the risk assessment of PrP^Sc^ infection among the PrP genotypes.

It remains unclear why M2C PrP^Sc^ lacked infectivity to the PrP-humanized mice despite the fact that they could propagate in the MM2C/MV2C patient brain. Although there was no evidence of successful transmission in the present study, very low infectivity of M2C PrP^Sc^ was reported in a transmission study using other PrP-humanized knock-in mouse lines [13]. The disease duration of MM2C/MV2C patients is the longest among sCJD subgroups [5], suggesting slow propagation of M2C PrP^Sc^. Therefore, M2C PrP^Sc^ may replicate less efficiently and take a longer time to propagate compared with the other PrP^Sc^ strains.

Dura mater graft-associated CJD with kuru plaques (p-dCJD) might be caused by infection of V2 PrP^Sc^ and/or Mi PrP^Sc^ to individuals with the 129M/M genotype. We reported previously that transmission of VV2 to animals with the 129M/M genotype caused p-dCJD like phenotype, *i.e.*, widespread PrP plaques and an accumulation of Mi PrP^Sc^, and that the transmission properties of p-dCJD were identical to those of VV2 [29]. It has been reported that the transmission properties of MVi + 2 were the same as those of VV2 [13,36]. In the present study, the transmission properties of MVi + 2C were also identical to those of VV2, and the transmission of MVi + 2 or MVi + 2C to animals with the 129M/M genotype caused p-dCJD like phenotype. These results suggest that p-dCJD is caused by infection of V2 PrP^Sc^ and/or Mi PrP^Sc^ from sCJD patients with VV2, MVi + 2, or MVi + 2C. Indeed, the incidence rate of p-dCJD among total dura mater graft-associated CJD is 32% [37], which is close to the sum total of the incidence of VV2 (15%), MVi + 2 (8%), and MVi + 2C (3%) in sCJD [5]. Since Mi PrP^Sc^ in individuals with the 129M/M genotype has never been observed in sCJD patients, it might be a characteristic fingerprint of the infection of V2 PrP^Sc^ and/or Mi PrP^Sc^ to the 129M/M individuals.

## Conclusions

The present study resolves the complicated pathogenesis of MV2. The phenotypic heterogeneity of MV2 stems from their different PrP^Sc^ origin(s).

## Competing interests

The authors declare that they have no competing interests.

## Authors’ contributions

AK, YI, MY, MY and TK performed histopathological analysis. AK and TK performed western blot analysis and drafted the manuscript. YI, HO, MY and MY clinically examined patients and collected tissue samples. YM and SM carried out transmission experiments. All authors read and approved the final manuscript.

## Supplementary Material

Additional file 1: Figure S1 Histotyping of the MV2 subgroups. (a-d) Histopathological features of MV2K (a), MV2K + C (b), MV2C (c), or MM2C (d). Hematoxylin and eosin stain and imunohistochemistry for PrP. Higher magnification of kuru plaques (arrows) is shown in the insets. Scale bar: 100 μm. (e-h) Regional distribution of PrP plaques in MV2K (e), MV2K + C (f), MV2C (g), or MM2C (h). Data are presented as mean ± SEM. FC, frontal cortex; TC, temporal cortex; OC, occipital cortex; BG, basal ganglia (putamen); TH, thalamus (dorsomedial nucleus); MB, midbrain; PO, pons; MO, medulla; CE, cerebellum (granular cell layer). Click here for file

Additional file 2: Figure S2 Immunohistochemical analysis of PrP in the brains of MM2C-inoculated PrP-humanized mice. *n*, number of mice positive for PrP accumulation in immunohistochemical analysis; *n*^*0*^, number of inoculated mice. G, grey matter; W, white matter. Scale bar: 100 μm. Click here for file
