# Supplementary material for: Deciphering the pathogenesis of sporadic Creutzfeldt-Jakob disease with codon 129 M/V and type 2 abnormal prion protein
Source: Acta Neuropathol Commun. 2013 Nov 13;1:74. doi: 10.1186/2051-5960-1-74 (PMC3833290; doi:10.1186/2051-5960-1-74)
Supplement: Additional file 1: Figure S1 — Histotyping of the MV2 subgroups. (a-d) Histopathological features of MV2K (a), MV2K + C (b), MV2C (c), or MM2C (d). Hematoxylin and eosin stain and imunohistochemistry for PrP. Higher magnification of kuru plaques (arrows) is shown in the insets. Scale bar: 100 μm. (e-h) Regional distribution of PrP plaques in MV2K (e), MV2K + C (f), MV2C (g), or MM2C (h). Data are presented as mean ± SEM. FC, frontal cortex; TC, temporal cortex; OC, occipital cortex; BG, basal ganglia (putamen); TH, thalamus (dorsomedial nucleus); MB, midbrain; PO, pons; MO, medulla; CE, cerebellum (granular cell layer). [file 2051-5960-1-74-S1.pdf]

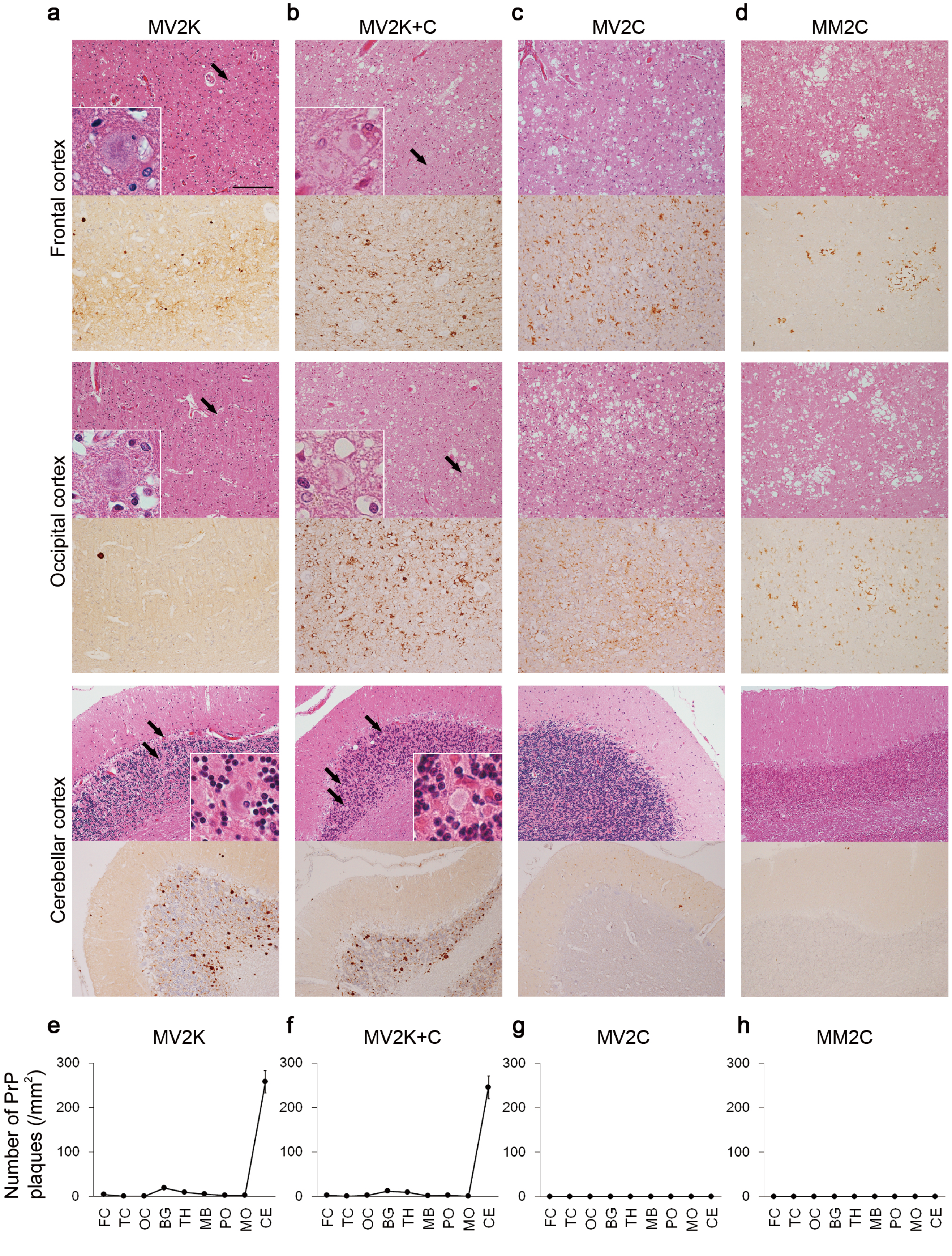

Supplementary Fig. 1

Histotyping of the MV2 subgroups. (**a**-**d**) Histopathological features of MV2K (**a**), MV2K+C (**b**), MV2C (**c**), or MM2C (**d**). Hematoxylin and eosin stain and imunohistochemistry for PrP. Higher magnification of kuru plaques (arrows) is shown in the insets. Scale bar: 100 µm. (**e**-**h**) Regional distribution of PrP plaques in MV2K (**e**), MV2K+C (**f**), MV2C (**g**), or MM2C (**h**). Data are presented as mean ± SEM. FC, frontal cortex; TC, temporal cortex; OC, occipital cortex; BG, basal ganglia (putamen); TH, thalamus (dorsomedial nucleus); MB, midbrain; PO, pons; MO, medulla; CE, cerebellum (granular cell layer).
