# Supplementary material for: Deciphering the pathogenesis of sporadic Creutzfeldt-Jakob disease with codon 129 M/V and type 2 abnormal prion protein
Source: Acta Neuropathol Commun. 2013 Nov 13;1:74. doi: 10.1186/2051-5960-1-74 (PMC3833290; doi:10.1186/2051-5960-1-74)
Supplement: Additional file 2: Figure S2 — Immunohistochemical analysis of PrP in the brains of MM2C-inoculated PrP-humanized mice. n, number of mice positive for PrP accumulation in immunohistochemical analysis; n0, number of inoculated mice. G, grey matter; W, white matter. Scale bar: 100 μm. [file 2051-5960-1-74-S2.pdf]

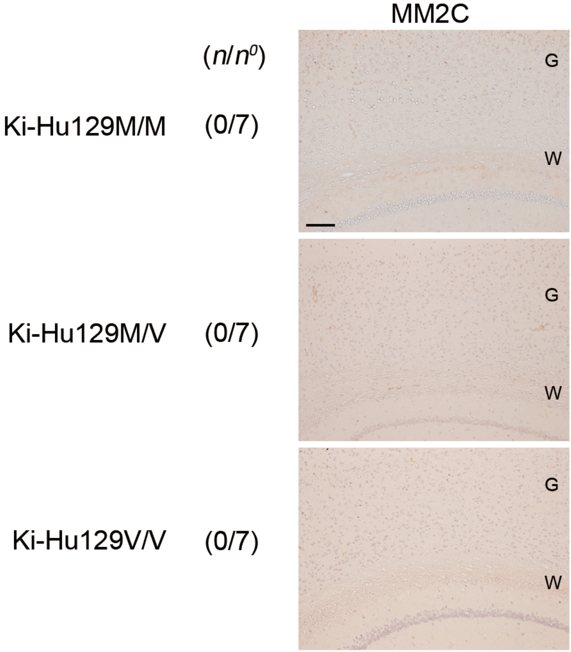

Supplementary Fig. 2

Immunohistochemical analysis of PrP in the brains of MM2C-inoculated PrP-humanized mice. *n*, number of mice positive for PrP accumulation in immunohistochemical analysis; n^0, number of inoculated mice. G, grey matter; W, white matter. Scale bar: 100 µm.
